# Inhalation of *Ortho*-Phthalaldehyde Vapor Causes Respiratory Sensitization in Mice

**DOI:** 10.1155/2011/751052

**Published:** 2011-07-14

**Authors:** Victor J. Johnson, Jeffrey S. Reynolds, Wei Wang, Kara Fluharty, Berran Yucesoy

**Affiliations:** ^1^Toxicology and Molecular Biology Branch, Health Effects Laboratory Division, National Institute for Occupational Safety and Health, Centers for Disease Control and Prevention, Morgantown, WV 26505-2888, USA; ^2^Pathology and Physiology Research Branch, Health Effects Laboratory Division, National Institute for Occupational Safety and Health, Centers for Disease Control and Prevention, Morgantown, WV 26505-2888, USA

## Abstract

*Ortho*-Phthalaldehyde (OPA) has been approved for high-level sterilization of heat-sensitive medical instruments and is increasingly being used as a replacement in the healthcare industry for glutaraldehyde, a known sensitizer. Numerous case reports have been published indicating workers and patients experiencing respiratory problems, anaphylaxis, skin reactivity, and systemic antibody production. Our laboratory previously demonstrated that OPA is a dermal sensitizer in mice. The goal of the present study was to determine if OPA is a respiratory sensitizer following inhalation exposure. Mice were exposed to OPA vapor and airway and lymph nodes were examined for cytokine gene expression and alterations in lymphocyte populations. Inhalation of OPA for 3 days resulted in a concentration-dependent increase in lymphocyte proliferation, mainly B lymphocytes, in the draining lymph nodes. A secondary challenge of mice with OPA resulted in a dramatic increase in the population of B lymphocytes expressing IgE. Expression of Th2 (IL-4, IL-5, and IL-13) and anti/proinflammatory (IL-10, TNF**α**, and IL-1**β**) cytokine genes was upregulated in the lymph nodes and the nasal mucosa. Mice exposed to the higher concentrations of OPA-produced OPA-specific IgG_1_ antibodies indicating systemic sensitization. These findings provide evidence that OPA has the potential to cause respiratory sensitization in mice.

## 1. Introduction

*Ortho*-Phthalaldehyde (OPA) is an aromatic dialdehyde, used as a high-level antimicrobial disinfectant for medical equipment which is sensitive to normal heat or steam sterilization processes, including endoscope, cystoscopes, and certain dental instruments. For 40 years, glutaraldehyde, another dialdehyde, has been the primary choice for disinfecting heat-sensitive medical devices; however, it has been reported to be a chemical sensitizer. Glutaraldehyde is known to have high affinity for biological amines, and its use as a tissue fixative capitalizes on this property. As such, glutaraldehyde and dialdehydes as a chemical class can bind to native proteins, thus, altering their presentation to the immune system. Haptenization of native proteins can lead to an aberrant immune response and the development of allergy. Several human studies have demonstrated the presence of IgE antibodies specific for glutaraldehyde adducts in the serum of exposed workers with respiratory disease [1, 2]. Importantly, workplace exposure to glutaraldehyde is known to induce occupational asthma [2–4] and allergic contact dermatitis [5] suggesting the need for safer alternatives. OPA has shown superior antimycobactericidal activity as compared to glutaraldehyde [6], allowing for its use at lower concentrations. In addition, low volatility and no need for activation have increased the use of OPA as a more practical alternative to glutaraldehyde. 

It is estimated that 3253 workers were potentially exposed to OPA compared to 376,330 for glutaraldehyde from 1981–1983 [7]. If OPA was fully adopted as an alternative for glutaraldehyde, it is a reasonable assumption that more than 300,000 US workers could now be exposed. The estimated use of OPA in 2002 was between 10,000 and 500,000 pounds [8]. OPA is commonly considered as a “safe” alternative to glutaraldehyde despite a paucity of information regarding the toxicity of this chemical and the potential health effects associated with exposure. Very few toxicology studies are available in the published literature to establish the safety of OPA. The few toxicity studies that have been performed suggest that OPA may be a chemical irritant and sensitizer and may act as an adjuvant for other allergens [9–12]. Currently there are no regulations regarding proper use and safe exposure levels of OPA in spite of the potential of exposure for a large number of healthcare workers and their patients. Concentrations of OPA ranging from 1.0 to 13.5 ppb have been detected in air samples collected from an endoscope cleaning unit of a hospital that used OPA as its primary disinfectant [13–15]. 

Several case reports have been presented in the literature questioning the safe substitution of OPA as a high-level sterilant in the healthcare industry. Fujita et al. [15] investigated a case involving a female nurse who exhibited slight dyspnea and dry cough that began a few months after switching to OPA for high-level sterilization in the endoscopy unit. The patient was subsequently diagnosed with bronchial asthma and experienced episodic attacks when working in the endoscopy unit. Another report identified four patients who experienced nine episodes of anaphylaxis with associated respiratory symptoms after a urology practice switched from using glutaraldehyde to OPA for cystoscope disinfection [16]. In a separate report, anaphylactic reactions with respiratory involvement occurred in two bladder cancer patients following repeated cystoscopic examination of their tumors [17] and a woman receiving repeated checkups by laryngoscopy [18, 19]. Two potential cases of occupational asthma in healthcare workers disinfecting endoscopes and similar devices with OPA have also been reported [20]. These case reports demonstrate that occupational and medical exposure to OPA can induce systemic anaphylaxis as well as pose a risk of respiratory sensitization.

Toxicity data derived from animal studies will be important for regulating and setting occupational exposure limits for OPA. Our laboratory recently demonstrated that mice dermally exposed to OPA tested positive in the local lymph node assay (LLNA) with associated increases in total and OPA-specific IgE levels, suggesting an IgE-mediated allergic mechanism [9]. The EC3 value for OPA was 0.051%, ten-fold lower than the working concentration for disinfection, establishing this chemical as a strong dermal sensitizer. The goal of the present studies was to determine the respiratory sensitization potential of inhalation exposure to OPA vapor. 

## 2. Methods

### 2.1. OPA Exposure System

In order to study the potential for the inhalation of OPA to cause respiratory sensitization, a nose-only exposure system was developed to minimize skin contact. The OPA exposure system consists of two major components, the vapor generator and the nose-only inhalation tower. Briefly, OPA (0–1000 ppb) was dissolved in ddH_2_O as a vehicle for delivery to the generator. The OPA generator consisted of a stainless steel septum T that was heated with a variable resistance heat rope to an internal air temperature of 105°C, a temperature that would ensure complete vaporization of the water vehicle. Air flow through the generator and exposure system was set at 10 LPM, and temperature of the system was controlled at 74 ± 2°C. This flow rate equated to 1 LPM/active exposure port on the nose-only tower and is sufficient to ensure adequate removal of exhalation gases to prevent dilution of the OPA atmosphere and rebreathing of respiratory gases, namely, CO_2_ [21]. A KDS100 syringe pump (KD Scientific Inc., Holliston, MA) was used to inject the OPA/vehicle (water) into the generator at a flow rate of 117 *μ*L/min conditioning the system air to 50 ± 3% relative humidity. Temperature and humidity were monitored using an HMP243 humidity/temperature transmitter (Vaisala Inc., Woburn, MA) placed in a nose-only restrainer to position the sensor in the breathing zone. The concentration of the OPA injection solution was adjusted to provide the desired OPA vapor concentration given the fixed injection rate of 117 *μ*L/min. The OPA/air mixture then entered a mixing chamber prior to the nose-only inhalation tower. The exposure atmosphere was pumped into the directed-flow nose-only exposure tower (InTox Products, Moriarty, MN). A vacuum was applied to the exhaust plenum to maintain a constant negative pressure of −0.1 inches water across the exposure tower controlled by a real-time electronic pressure controller (Alicat Scientific, Tucson, AZ) that monitored and dynamically adjusted the pressure within the exhaust plenum. This minimized leaks to atmosphere as well as ensured sufficient removal of respiratory gases from the breathing zone. Mice were exposed to OPA using nose-only restrainers. The calculated OPA vapor concentration was confirmed empirically using a laboratory assay based upon the fluorescence of OPA-protein conjugates. Air samples from the inhalation tower were sampled from the breathing zone of a free exposure port using a midget impinger. The sample was used to detect a fixed concentration of L-alanine as the amine acceptor, and the OPA concentration was determined relative to an OPA standard curve monitored at 442 nm. 

### 2.2. Experimental Animals

**Figure 1:**
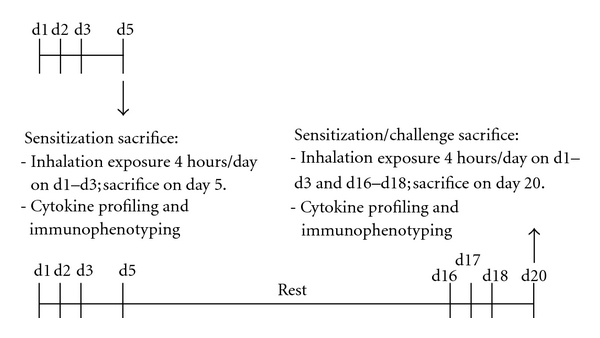
OPA inhalation and sacrifice schedule.

Female specific-pathogen-free inbred C57BL/6 mice were purchased from Jackson Laboratories (Bar Harbor, ME) at 6 to 7 weeks of age. Upon arrival, the mice were quarantined for 2 weeks and acclimated to a 12-hour light/dark cycle. Animals were housed in ventilated microisolator cages in environmentally controlled conditions at NIOSH animal facilities in compliance with AAALAC-approved guidelines and an approved IACUC protocol. The animal rooms were monitored for specific pathogens through disease surveillance and a sentinel animal program. Food and water were provided *ad libitum*. Mice were randomized across two exposure paradigms (Figure 1) each with a control (filtered and conditioned air) and four concentrations of OPA (125–1000 ppb). The sensitization exposure paradigm involved 4-hour inhalation exposures (4 hours/exposure) on day 1–3 followed by euthanasia 48 hours after final exposure. The sensitization/challenge exposure paradigm consisted of inhalation exposure on days 1–3 and again on days 16–18 followed by sacrifice 48 hours later. The exposure environment was maintained at 74 ± 2°C and 50 ± 3% relative humidity.

### 2.3. Tissue Collection

 Mice were sacrificed via pentobarbital overdose (200 mg/kg, i.p.) 48 hours following the final exposure. Blood was collected from the abdominal aorta and serum was isolated and frozen at −80°C until assessment of antibody production. For gene expression analysis, the head was removed and the nasal cavity was flushed with RNAlater (Qiagen, Valencia, CA), the skin and fur, top of the skull, brain and lower jaw were removed and the remaining tissue was stored in 10 volumes of RNAlater at 4^*º*^C until dissection and tissue removal (3 days). The left mandibular lymph nodes and the lungs were removed, lungs were inflated with RNAlater, and then the tissues were stored in 10 volumes of RNAlater at −20°C until RNA extraction for PCR analysis. The nasal cavity was opened by removing the nasal bones and flattening the skull along the anterior-posterior axis. Using blunt dissection in RNAlater, the nasal mucosa lining the maxilloturbinates and lateral wall were removed as a single sample and stored at −20°C in RNAlater until processed for microarray and PCR gene expression analysis. The right mandibular lymph nodes were removed and placed in 3 mL PBS at 4°C and quickly processed for flow cytometric phenotyping as described below.

### 2.4. OPA-Specific Antibody Detection

OPA-specific immunoglobulin G_1_ (IgG_1_) and IgE serum antibodies were detected using an ELISA procedure as previously described [9]. Briefly, Immulon-4 microtiter plates (Nunc, Thermo Scientific) were coated overnight at 4°C with mouse serum albumin (MSA; 10 *μ*g/mL in carbonate buffer, pH 9.5). Plates were washed 3 times with 0.05 M borate buffer followed by the addition of 0.5% OPA in distilled deionized water for 1 hour at room temperature. Plates were washed 3 times with PBS/0.05% Tween-20 wash buffer, and nonspecific binding sites were blocked with 1% BSA in PBS/0.05% Tween-20 for 30 minutes. A two-fold dilution series (1/10 to 1/5120) of serum was added to wells coated with MSA-only and wells with OPA-conjugated MSA and incubated for 2 hours at 4°C. Plates were washed 3 times with PBS/0.05% Tween-20, and then biotin-conjugated antibodies specific for mouse IgG_1_ or IgE (BD Biosciences, San Jose, CA) were added for 1 hour at 4°C. Finally, plates were washed 4 times, and avidin-HRP was added for 30 minutes at room temperature followed by 4 washes. TMB-Turbo substrate (Thermo Fisher Scientific, Pierce, Rockford, IL) was added for 30 minutes followed by the addition of 2 M H_2_SO_4_ stop solution. Absorbance was read on a SpectraMax plate reader (Molecular Devices, Sunnyvale, CA) at 650 nm during color development and at 450 nm following addition of stop solution. 

### 2.5. Assessment of Nasal Mucosa Gene Expression

Total cellular RNA was extracted from the nasal mucosa using the Qiagen RNeasy kit (Qiagen, Valencia, CA) according to the manufacturer's instructions following homogenization in RLT buffer using a TissueLyser II (Qiagen, Valencia, CA) bead mill system. One *μ*g of RNA was reverse-transcribed using random hexamers and 60 U of Superscript II (Life Technologies, Grand Island, NY). Real-time PCR primer/probe sets for murine IL-1*β*, IL-4, IL-5, IL-13, IFN*γ*, TNF*α*, and 18s were purchased from Applied Biosystems (Foster City, CA). Real-time PCR was performed using Taqman Universal Master mix with Amperase in an Applied Biosystems 7900HT (Applied Biosystems, Foster City, CA) for 1 cycle at 50°C for 2 minutes, and 95°C for 10 minutes, followed by 60 cycles at 95°C for 15 seconds and 60°C for 1 minute. Relative differences in mRNA expression between control and treatment groups were determined by the relative quantification method developed by Pfaffl [22]. This method uses gene specific PCR efficiencies to more accurately generate relative changes based on threshold cycle. Target gene expression was normalized to the housekeeping gene 18s/rRNA. 

### 2.6. Flow Cytometric Phenotyping of Draining Lymph Nodes

All antibodies and isotype controls for phenotyping T and B lymphocytes were purchased from BD Pharmingen (San Jose, CA). The right mandibular lymph nodes that drain the nasal mucosa were collected in 3 mL PBS and dissociated using the frosted ends of two microscope slides. Cell counts were performed using a Coulter Counter (Z2 model, Beckman Coulter, Brea, CA), and 1 × 10^6^ cells per sample were added to the wells of a 96-well plate. Cells were washed using staining buffer (0.2% bovine serum albumin/0.1% sodium azide in DPBS; BD Pharmingen, San Jose, CA) and then incubated for 10 minutes with Fc block (clone 2.4G2). The cells were then incubated with anti-CD3 (APC, clone 145-2C11)/anti-CD4 (FITC, clone RM4-5)/anti-CD8 (PE, clone 53-6.7) or anti-CD45RA/B220 (PE, clone RA3-6B2)/anti-IgE antibodies (FITC, clone R-35-72) or the appropriate isotype controls diluted in staining buffer for 30 minutes. Cells were washed, incubated with propidium iodide (PI) for 5 minutes to stain dead cells. After a final wash, cells were resuspended in staining buffer and analyzed with a FACSCaliber flow cytometer (Becton Dickinson, San Jose, CA) using a PI viability gate. Data for a total of 10,000 cells were collected based on the forward-side scatter lymphocyte gate. 

### 2.7. Statistical Analysis

The OPA inhalation exposure system is capable of exposing mice to a single concentration with an equivalent inhalation tower used for the concurrent control group. Therefore, all concentrations for each exposure paradigm (Figure 1) required its own concurrent control, and all data analysis was performed relative to the concurrent control. Treatment effects were determined using a Student's *t*-test comparing a single OPA concentration to the concurrent control. Differences were considered significant at *P* < .05. For clarity of presentation, data have been normalized to the concurrent control group and shown as fold change, unless otherwise stated.

## 3. Results

### 3.1. OPA Inhalation Did Not Induce Overt Signs of Systemic Toxicity and Respiratory Distress

Inhalation exposure to OPA did not cause observable clinical signs of systemic toxicity or respiratory distress throughout the exposure dose response. All mice actively groomed following each exposure period and were observed to eat and drink shortly after exposure. However, mice treated with 1000 ppb OPA for 3 days followed by sacrifice on the 5th day (sensitization exposure; Figure 1) had lost approximately 10% of their initial body weight versus 1.9% in the controls. Over the rest period, mice regained weight at a similar rate as the control group. Subsequent inhalation challenge with the same OPA concentration (sensitization/challenge exposure; Figure 1) resulted in a smaller body weight loss (5% loss from initial body weight). Similar body weight loss was observed in mice exposed to 500 ppb OPA but concentrations below 500 ppb showed similar body weight changes as observed in the control mice (data not shown). No mice died from OPA inhalation prior to the scheduled study termination.

### 3.2. Inhalation Exposure to OPA Vapor Induced the Production of OPA-Specific Antibodies

Serum was collected and evaluated to determine if mice exposed to OPA developed systemic sensitization resulting in the production of antibodies specific for OPA-conjugated protein. No OPA-specific antibodies were observed in any of the control mice or mice exposed to OPA using the sensitization exposure. In contrast, inhalation challenge of mice to ≥500 ppb OPA vapor resulted in OPA-specific IgG_1_ production. The OD_450_ values were 1.76 ± 0.02 and 1.35 ± 0.35 for a 1/20 dilution of serum from mice treated with 500 or 1000 ppb OPA, respectively, versus a background OD_450_ in the control sera of 0.086 ± 0.02. Serum from mice treated with 500 and 1000 ppb OPA also showed mild antibody specificity for native MSA as observed in our previous studies following dermal exposure to OPA [9]. OPA-specific IgE was not detected in the serum of any mice (data not shown).

### 3.3. OPA Inhalation Induces Cytokine Gene Expression in the Airways and Draining Lymph Nodes

**Figure 2:**
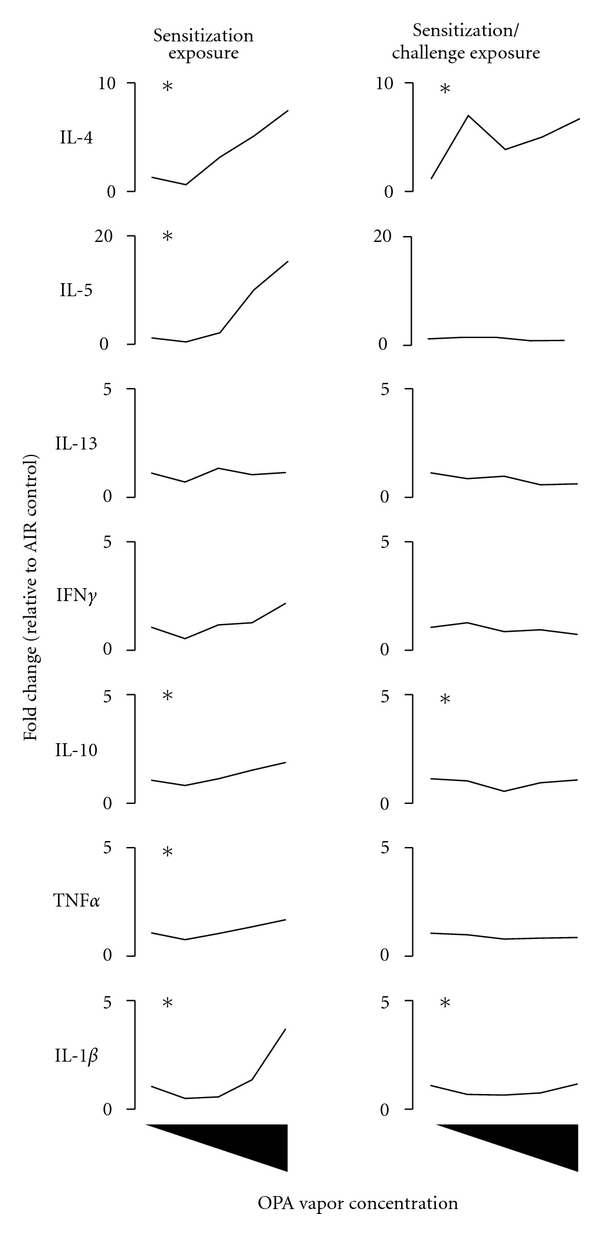
*Inhalation of OPA vapor induces the expression of Th2 and pro/anti-inflammatory cytokines in the draining lymph nodes of mice. *Mice were exposed to OPA (125, 250, 500, 1000 ppb) or filtered air according to the schedule shown in Figure 1. Two days following the final exposure, the mandibular lymph nodes from the left side of the neck were removed and processed for gene expression analysis. Data are presented as mean (*n* = 5) and represent fold change relative to the concurrent control group. *Indicates that the cytokine was significantly increased at one or more of the OPA concentrations. Refer to Table S1 of the supplementary material available online at doi: 10.1155/2011/751052 for the empirical gene expression data.

Gene expression analysis of key cytokine genes can provide insight into the immunotoxicity of inhaled chemicals especially when examined in the target mucosal tissue lining the airways and the associated draining lymph nodes. Pro/anti-inflammatory cytokines (IL-10, TNF*α*, IL-1*β*) are important in the initial stages of an immune response to a chemical sensitizer and can shape the developing immune response towards Th1 immunity (IFN*γ*), Th2 immunity (IL-4, IIL-5, IL-13), or a combination. Sensitization and allergy are supported mainly by Th2 cytokine responses although Th1 cytokines have been shown to be important for chemical sensitizers [23]. Lymph nodes in the cervical region drain the skin of the head and the nasal mucosa. Since OPA is a highly reactive chemical, the mandibular lymph nodes draining the nasal mucosa, a target site, were examined for gene expression changes. The inhalation of OPA during sensitization resulted in concentration-dependent upregulation of IL-4 and IL-5 expression (significant increases ≥500 ppb OPA; Table S1) in the mandibular lymph nodes (Figure 2). The expression of TNF*α*, IL-1*β*, and IL-10 were also increased in the lymph nodes of mice treated with 1000 ppb OPA. Subsequent OPA challenge for an additional 3 days resulted in increased IL-4 gene expression throughout the concentration range tested (Figure 2; Table S1). The expressions of IL-10 and IL-1*β* were also increased in the lymph nodes following challenge with OPA although to a lesser extent than following sensitization alone (Figure 2; Table S1).

**Figure 3:**
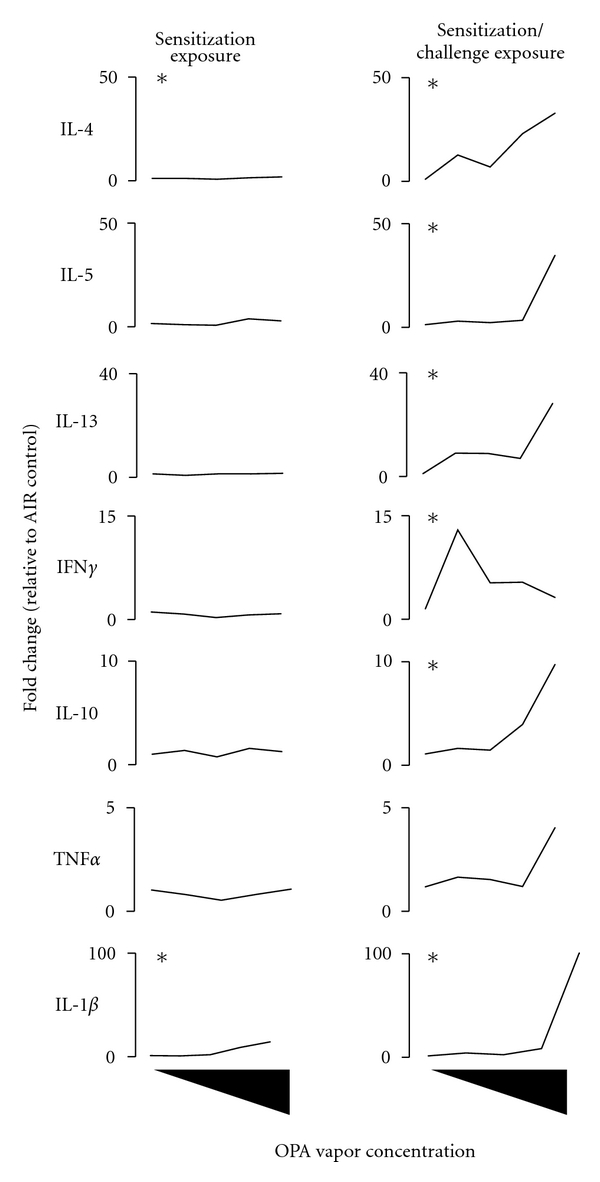
*Inhalation of OPA vapor induces the expression of Th2, Th1, and pro/anti-inflammatory cytokines in the nasal mucosa of mice. *Mice were exposed to OPA (125, 250, 500, 1000 ppb) or filtered air according to the schedule shown in Figure 1. Two days following the final exposure, the mucosal tissue lining the maxilloturbinates and lateral wall of the nasal cavity were removed and processed for gene expression analysis. Data are presented as mean (*n* = 5) and represent fold change relative to the concurrent control group. *Indicates that the cytokine was significantly increased at one or more of the OPA concentrations. Refer to Table S1 for the empirical gene expression data.

**Figure 4:**
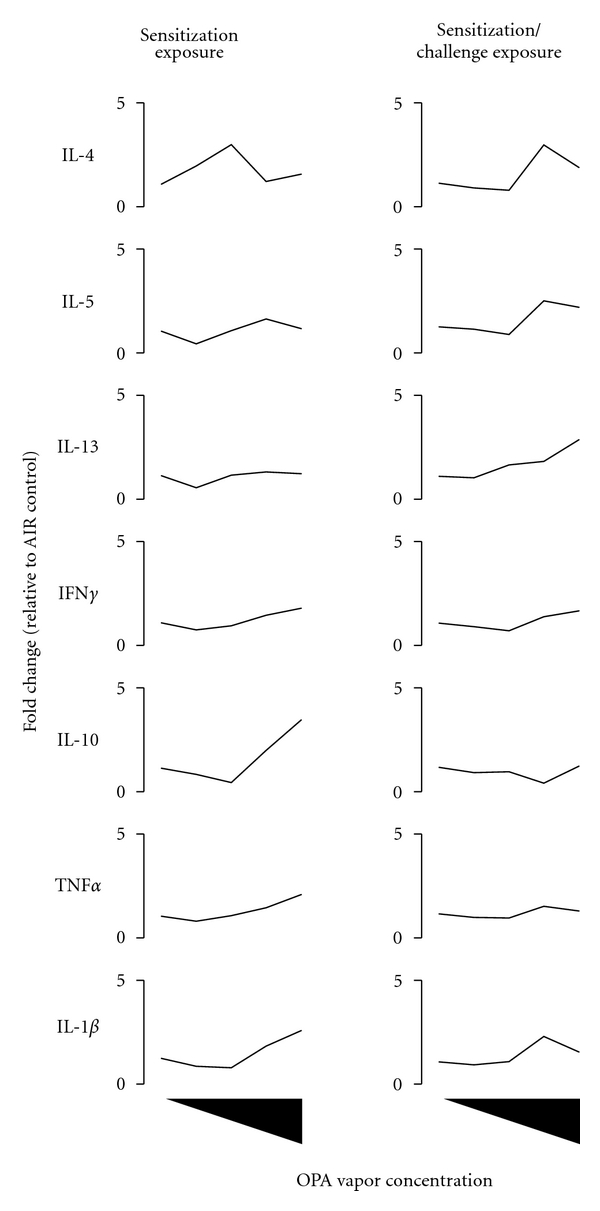
*Effect of respiratory exposure to OPA vapor on the expression of Th2, Th1, and pro/anti-inflammatory cytokines in the lungs of mice. *Mice were exposed to OPA (125, 250, 500, 1000 ppb) or filtered air according to the schedule shown in Figure 1. Two days following the final exposure, the lungs were removed, inflated with RNALater, and processed for gene expression analysis. Data are presented as mean (*n* = 5) and represent fold change relative to the concurrent control group. *Indicates that the cytokine was significantly increased at one or more of the OPA concentrations. Refer to Table S1 for the empirical gene expression data.

The inhalation of OPA also increased cytokine gene expression in the mucosal tissue lining the upper airways (Figure 3; Table S1). Increased IL-4 expression in mice exposed to 1000 ppb OPA and a concentration-dependent stimulation of IL-1*β* in the nasal mucosa occurred in mice exposed to OPA during sensitization only suggesting acute inflammation. Challenging mice after an 11-day rest period to the same concentration of OPA vapor for an additional 3 days caused marked concentration-dependent increases in all of the cytokine genes interrogated. IL-4, IL-5, and IL-1*β* were upregulated at all concentrations of OPA while IL-13, IFN*γ*, TNF*α*, and IL-10 were increased at ≥500 ppb. Mice that inhaled the higher concentrations of OPA also showed increased expression of IL-4, IL-5, IL-13, and IL-1*β* in the lung tissues although not statistically significant (Figure 4; Table S1).

### 3.4. Respiratory Exposure to OPA Induces Lymphocyte Proliferation and an Allergic Phenotype in the Mandibular Lymph Nodes

**Figure 5:**
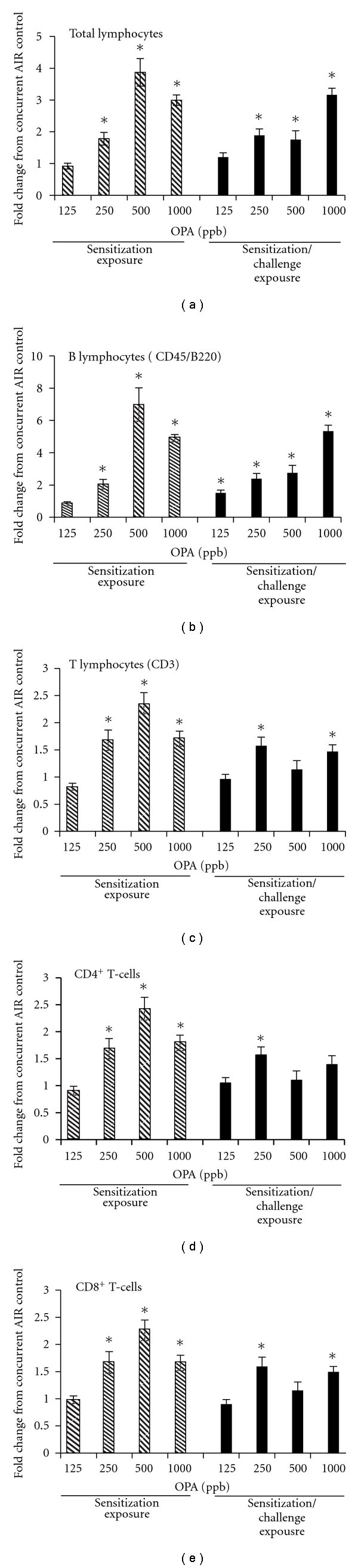
*OPA inhalation stimulates B- and T-lymphocyte proliferation in the draining lymph nodes of mice. *Mice were exposed to OPA or filtered air according to the schedule shown in Figure 1. Two days following the final exposure, the mandibular lymph nodes from the right side of the neck were removed and immediately processed into single-cell suspensions. Scatter properties were used to identify lymphocytes followed by fluorescent antibodies labeling to identify B lymphocytes, T lymphocytes (total, CD4^+^ and CD8^+^) using flow cytometry. Data are presented as fold change relative to the concurrent control. Statistical analysis was performed on the absolute cell counts for each lymphocyte population. Hatched bars represent mice that were exposed for 3 days followed by sacrifice on day 5 (sensitization exposure, Figure 1). Solid bars represent mice that were exposed on d1–d3, d16–d18 and sacrificed on d20 (sensitization/challenge exposure, Figure 1). Mean ± SEM (*n* = 5). *Significantly different from concurrent control group at *P* < .05. Absolute cell counts are presented in Table S2.

A hallmark characteristic of respiratory allergy is proliferation of lymphocytes, namely, B lymphocytes in the lymph nodes draining the target tissue. OPA inhalation resulted in a concentration-dependent increase in total lymphocytes in the mandibular lymph nodes with a maximum increase of approximately 4 fold over the control mice (Figure 5). Both T lymphocytes and B lymphocytes were increased in the lymph nodes of OPA-exposed mice, however, the increase in B lymphocytes was much greater. The ratio of T : B lymphocytes was reduced in a concentration-dependent manor in mice treated with OPA reflecting the greater proliferation of B lymphocytes relative to T lymphocytes (Table 1). The ratio of CD4:CD8 T lymphocytes in the draining lymph nodes did not change in any of the treatment groups relative to the controls (data not shown).

### 3.5. OPA Inhalation Induces Isotype Switching to IgE^+^ B Lymphocytes in the Draining Lymph Nodes

**Figure 6:**
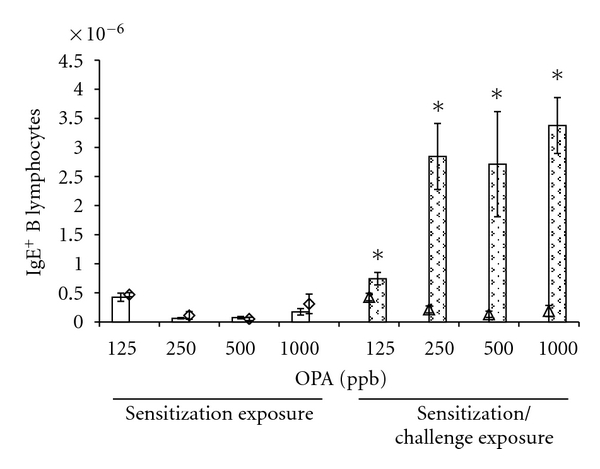
*OPA inhalation induces isotype switching in B lymphocytes to IgE. *Mice were exposed to OPA or filtered air according to the schedule shown in Figure 1. Two days following the final exposure, the mandibular lymph nodes from the right side of the neck were removed and immediately processed into single-cell suspensions. Scatter properties were used to identify lymphocytes followed by fluorescent antibodies labeling to identify B lymphocytes using flow cytometry. Fluorescent anti-IgE antibodies were used to identify B-lymphocyte expressing IgE on their membrane. Open bars and open diamonds represent mice that were exposed OPA or filtered air, respectively, for 3 days followed by sacrifice on day 5 (sensitization exposure, Figure 1). Stippled bars and open triangles represent mice that were exposed OPA or filtered air, respectively, on d1–d3, d16–d18 and sacrificed on d20 (sensitization/challenge exposure, Figure 1). Mean ± SEM (*n* = 5). *Significantly different from concurrent control group at *P* < .05.

An important characteristic of an allergic response is the isotype switch to IgE producing B lymphocytes. We utilized multicolor flow cytometry to identify the B lymphocyte population in the draining lymph nodes and enumerated the IgE^+^ and IgE^−^ populations using an anti-IgE antibody.  Figure 6 shows that inhalation exposure to OPA during sensitization only did not induce isotype switching to IgE^+^ B lymphocytes at any concentration tested. In contrast, inhalation challenge 11 days later to the same OPA concentration resulted in a marked increase in the number of B lymphocytes expressing IgE indicating isotype switching to IgE (Figure 6). The percentage of total B-lymphocytes producing IgE in the mandibular lymph nodes increased from 7.32 ± 1.08% in the control group to 11.38 ± 0.99, 34.01 ± 5.06, 41.62 ± 8.72, and 22.67 ± 2.09% in mice exposed to 125, 250, 500, and 1000 ppb OPA, respectively. These effects were significant at all concentrations of OPA tested.

## 4. Discussion

*Ortho*-Phthalaldehyde has been approved by the FDA for the high-level disinfection of reusable heat sensitive medical/dental instruments [24] and is increasingly being substituted for glutaraldehyde in the healthcare industry. Approval was rendered despite a lack of toxicity data supporting OPA as a safe alternative. There have been numerous case reports on skin and respiratory complications in healthcare workers and patients exposed to OPA suggesting similar health risks to glutaraldehyde. The goal of the present studies was to determine if OPA has the potential to act as a respiratory sensitizer following inhalation exposure. Our previous studies demonstrated that OPA was a potent dermal sensitizer following skin exposure [9]. In order to study the potential for the inhalation of OPA to cause respiratory sensitization, a nose-only exposure system was developed to minimize skin contact. Cytokine gene expression and lymphocyte phenotyping in the respiratory mucosa and draining lymph nodes provide evidence that OPA is a respiratory sensitizer in mice.

OPA is a highly reactive chemical with an affinity for biological amines, a property exploited in its use as a biocidal agent. This property is also exploited for the detection of biomolecules since upon interaction with amines, OPA becomes highly fluorescent [25]. By virtue of its ability to react with proteins, OPA may also act as a hapten in biological systems and could possibly lead to aberrant immune and allergic reactions. These hapten reactions form the basis for sensitization resulting from exposure to reactive low molecular weight chemicals in the workplace. The present work shows that the inhalation of OPA results in the systemic production of IgG_1_ antibodies that are specific for OPA-MSA, supportive evidence that OPA-protein conjugates formed *in vivo* are immunogenic. The production of IgG_1_ in by B lymphocytes requires support from CD4^+^ T lymphocytes producing IL-4 and IL-5 which facilitate isotype switching and maturation, respectively [26]. Passive transfer of chemical-specific IgG antibody to naïve mice has been shown to induce respiratory symptoms following exposure to toluene diisocyanate [27] and trimellitic anhydride [28] indicating a potential role for IgG in the pathogenesis of chemical-induced airway allergy. It is also possible that OPA-specific IgG_1_ may represent a marker of exposure as has been suggested for diisocyanates [29, 30]. 

OPA-specific IgE was not detected following inhalation exposure; however, aggressive dermal exposure of mice to OPA resulted in the production of OPA-specific IgG_1_, IgG_2a_, and IgE isotypes supporting an allergic immune response to this chemical [9]. Similar chemical-hapten-specific antibody responses have been observed in rodent models for other low molecular weight chemicals including toluene diisocyanate [23, 31] and trimelitic anhydride [32]. OPA-specific antibodies have been detected in the serum of healthcare workers experiencing respiratory and dermal symptoms resulting from workplace exposure to OPA [18]. The presence of OPA-specific antibodies in the serum of sensitized workers may result from dermal and/or airway exposure. Importantly, basophils from healthcare workers were shown to have bound OPA-specific IgE as *in vitro* culture with 0.55% OPA solution resulted in histamine release that was similar to treating the cells with anti-IgE antibody [18]. This demonstrates a functional importance of OPA-specific antibodies. 

A hallmark feature of chemical sensitizers is a positive reaction in the local lymph nodes assay (LLNA). Our laboratory showed that OPA tested positive in the LLNA with an EC3 of 0.051% [9]. This suggests that OPA is a powerful dermal sensitizer and may cause health risks when in contact with the skin at concentrations as much as 10 fold below the effective working concentration (0.55%) for disinfecting medical devices. Recently, a similar approach to the dermal LLNA has been presented for the identification of respiratory sensitizers. The respiratory LLNA utilizes inhalation as the route of exposure and examines the lymph nodes that drain the nasal mucosa for lymphocyte proliferation [33] and cytokine production [34]. Although the traditional LLNA endpoint of H^3^ incorporation as a metric of lymphocyte proliferation was not used in the present study, mice that were exposed to OPA by inhalation showed a concentration-dependent increase in the absolute and relative lymphocyte counts in the mandibular lymph nodes. This response was significant at concentrations ≥250 ppb OPA following the 3 days of inhalation as well as in mice that were reexposed for an additional 3 days. B lymphocytes accounted for the majority of the proliferation suggesting a shift towards a humoral immune response. Although T-lymphocyte numbers also increased in the mandibular lymph nodes, the ratio of CD4 : CD8 T cells remained unchanged (data not shown). 

Isotype switching to IgE is a cardinal feature of a type I hypersensitivity response. The serum of mice exposed to OPA via inhalation was negative for IgE antibodies specific for OPA-MSA although our previous work showed that dermal exposure to OPA did induce OPA-specific IgE [9]. The lack of systemic IgE may be due to the sensitivity of the ELISA assay or may result from a less aggressive sensitization response following inhalation exposure relative to dermal. Phenotyping the B-lymphocyte population in the draining lymph nodes can provide clues to the isotype specificity of the proliferating population. It has been shown that B lymphocytes from the lymph nodes of mice exposed to Th2 respiratory sensitizers are positive for IgE on their plasma membrane [35–38]. The inhalation of OPA vapor during sensitization did not increase the population of IgE^+^ B lymphocytes at any concentration tested despite the dramatic proliferation observed for the B-lymphocyte population. In contrast, there was a significant increase in the number of IgE^+^ B-lymphocytes in the mandibular lymph nodes at all concentrations of OPA following OPA challenge. Nearly 50% of the B lymphocytes from mice exposed to 500 ppb OPA were positive for IgE indicating isotype switching to an allergic phenotype, evidence that OPA may cause type I hypersensitivity. 

Isotype switching to IgE is regulated by Th2 cytokines, namely, IL-4 [39] which is produced primarily by CD4^+^ T lymphocytes [40]. There was increased proliferation of T lymphocytes following the inhalation of OPA, and these cells likely play an important role in shaping the humoral immune response to this chemical. The inhalation of OPA resulted in a concentration-dependent increase in IL-4 and IL-5 gene expression in the mandibular lymph nodes even following sensitization exposure regimen. Mice that were challenged with OPA showed significant upregulation of IL-4 gene expression at all concentrations of OPA supporting class switching to IgE in the B-lymphocyte population. In contrast, Th2 cytokine gene expression was unaltered in the nasal mucosa and lung following sensitization exposure. Inhalation challenge with OPA caused a marked concentration-dependent increase in the expression of the Th2 cytokine IL-4, IL-5, and IL-13 and to a lesser degree, the Th1 cytokine IFN*γ*. This strong shift towards Th2 cytokine production in the target mucosal tissue further supports an allergic response to OPA inhalation. In addition to these cytokines, the proinflammatory cytokine IL-1*β* was upregulated and is known to play an important role in priming antigen presenting cells. Th2 cytokine gene expression in the lungs was also upregulated in mice that received the challenge exposure to OPA although not statistically significant. The ability of OPA to stimulate Th2 and proinflammatory cytokines may be one mechanism responsible for its adjuvant-like properties observed in a murine OVA allergy model [11].

Overall, the findings of this study demonstrate that OPA has the potential to induce respiratory sensitization following inhalation exposure. Characteristics of the immune response to OPA suggest that exposure to this chemical may cause a type I hypersensitivity response as there was a large increase in the IgE^+^ B-lymphocyte population. The immune response to OPA is similar to other low and high molecular weight chemicals that are known to cause occupational rhinitis and asthma, indicating the need for the regulation of OPA in the workplace. The concentration range tested in the present study is at least 10 fold higher than air concentrations that can be found in the workplace [14]. Therefore, further studies with lower concentrations and longer exposure periods are important to better characterize the immunotoxicity of OPA and to facilitate risk assessment. Additionally, the understanding the interaction between skin and respiratory exposure will be important for risk assessment for OPA. Previous work with other low molecular weight sensitizers have shown that skin exposure can prime the immune system to respond to future airway exposures [41–44]. There is a high potential for skin exposure during manual reprocessing of medical devices with OPA. The potent sensitizing response to dermal exposure [9] may significantly lower the threshold air concentration of OPA required to elicit an immune response in the airways. Our laboratory is currently examining the interaction between skin and airways in the development of OPA-induced allergic respiratory disease using the mouse model.

## Supplementary Material

Numerical data for cytokine mRNA expression levels (Table S1) and lymph node immunophenotyping (Table S2). Mice were exposed to OPA (125, 250, 500, 1000 ppb) or filtered air according to the schedule shown in Figure 1. Two days following the final exposure, the mandibular lymph nodes (left side), nasal mucosa and lungs were removed and processed for gene expression analysis (Table S1). Lymph nodes from the right side were removed and processed into single cell suspensions and labeled for immunophenotyping according to the Methods section (Table S2).

## Figures and Tables

**Table 1:** Inhalation of OPA skews the lymphocyte population heavily towards B lymphocytes in the mandibular lymph nodes.

OPA (ppb)	T-lymphocyte/B-lymphocyte Ratio
	(Fold change from concurrent control)
Sensitization Exposure	
125	1.19 ± 0.09^a^
250	−1.41 ± 0.19
500	−3.22 ± 0.24*
1000	−3.20 ± 0.29*

Sensitization/Challenge Exposure	
125	−1.58 ± 0.08*
250	−1.56 ± 0.11*
500	−2.39 ± 0.29*
1000	−4.13 ± 0.20*

*Significantly different from concurrent control group at *P* < .05.

^a^Mean ± SEM (*n* = 5).
