# Supplementary material for: Inhalation of Ortho-Phthalaldehyde Vapor Causes Respiratory Sensitization in Mice
Source: J Allergy (Cairo). 2011 Jul 14;2011:751052. doi: 10.1155/2011/751052 (PMC3137992; doi:10.1155/2011/751052)
Supplement: Supplementary file 1 — Numerical data for cytokine mRNA expression levels (Table S1) and lymph node immunophenotyping (Table S2). Mice were exposed to OPA (125, 250, 500, 1000 ppb) or filtered air according to the schedule shown in Figure 1. Two days following the final exposure, the mandibular lymph nodes (left side), nasal mucosa and lungs were removed and processed for gene expression analysis (Table S1). Lymph nodes from the right side were removed and processed into single cell suspensions and labeled for immunophenotyping according to the Methods section (Table S2). [file 751052.f1.docx]

Table S1: Gene expression data shown as fold change relative to the concurrent control group.

| **ppb** | **IL-4** | **IL-5** | **IL-13** | **IFNγ** | **TNFα** | **IL-1β** | **IL-10** |
| --- | --- | --- | --- | --- | --- | --- | --- |
| Lymph node gene expression: Sensitization Exposure | | | | | | | |
| 0* | 1.3 ± 0.49^a^ | 1.16 ± 0.32 | 1.11 ± 0.24 | 1.05 ± 0.17 | 1.06 ± 0.18 | 1.03 ± 0.13 | 1.05 ± 0.18 |
| 125 | 0.61 ± 0.14 | 0.45 ± 0.17 | 0.7 ± 0.33 | 0.53 ± 0.14 | 0.76 ± 0.13 | 0.49 ± 0.12 | 0.81 ± 0.09 |
| 250 | 3.14 ± 0.57 | 2.12 ± 0.9 | 1.33 ± 0.28 | 1.16 ± 0.18 | 1.04 ± 0.13 | 0.56 ± 0.04 | 1.13 ± 0.13 |
| 500 | 5.09 ± 0.75 | 9.99 ± 1.32 | 1.04 ± 0.25 | 1.26 ± 0.1 | 1.35 ± 0.22 | 1.35 ± 0.34 | 1.52 ± 0.26 |
| 1000 | 7.44 ± 0.62 | 15.27 ± 1.08 | 1.13 ± 0.14 | 2.15 ± 0.65 | 1.68 ± 0.19 | 3.67 ± 0.67 | 1.87 ± 0.19 |
| Lymph node gene expression: Sensitization/Challenge Exposure | | | | | | | |
| 0 | 1.18 ± 0.33 | 1.17 ± 0.33 | 1.12 ± 0.3 | 1.05 ± 0.16 | 1.06 ± 0.18 | 1.08 ± 0.22 | 1.12 ± 0.26 |
| 125 | 6.98 ± 1.09 | 1.44 ± 0.12 | 0.86 ± 0.17 | 1.26 ± 0.13 | 0.98 ± 0.15 | 0.67 ± 0.07 | 1.02 ± 0.04 |
| 250 | 3.86 ± 1.16 | 1.47 ± 0.38 | 0.96 ± 0.11 | 0.85 ± 0.1 | 0.79 ± 0.11 | 0.64 ± 0.03 | 0.55 ± 0.05 |
| 500 | 4.99 ± 0.77 | 0.87 ± 0.27 | 0.57 ± 0.15 | 0.93 ± 0.04 | 0.82 ± 0.1 | 0.75 ± 0.03 | 0.93 ± 0.09 |
| 1000 | 6.67 ± 1.24 | 0.9 ± 0.3 | 0.62 ± 0.08 | 0.72 ± 0.13 | 0.85 ± 0.14 | 1.14 ± 0.12 | 1.07 ± 0.16 |
| Nasal mucosa gene expression: Sensitization Exposure | | | | | | | |
| 0 | 1.08 ± 0.19 | 1.63 ± 0.46 | 1.27 ± 0.33 | 1.12 ± 0.26 | 1.03 ± 0.13 | 1.05 ± 0.14 | 1.02 ± 0.1 |
| 125 | 1.09 ± 0.58 | 0.97 ± 0.43 | 0.74 ± 0.2 | 0.83 ± 0.2 | 0.8 ± 0.18 | 0.83 ± 0.18 | 1.39 ± 0.21 |
| 250 | 0.77 ± 0.2 | 0.67 ± 0.2 | 1.36 ± 0.55 | 0.29 ± 0.14 | 0.53 ± 0.08 | 2.13 ± 0.51 | 0.77 ± 0.27 |
| 500 | 1.38 ± 0.32 | 3.76 ± 1.42 | 1.3 ± 0.49 | 0.7 ± 0.16 | 0.81 ± 0.09 | 9.02 ± 2.9 | 1.58 ± 0.45 |
| 1000 | 1.81 ± 0.12 | 2.84 ± 0.8 | 1.53 ± 0.54 | 0.86 ± 0.23 | 1.07 ± 0.07 | 14.31 ± 3.46 | 1.27 ± 0.2 |
| Nasal mucosa gene expression: Sensitization/Challenge Exposure | | | | | | | |
| 0 | 1.1 ± 0.16 | 1.06 ± 0.17 | 1.04 ± 0.14 | 1.22 ± 0.38 | 1.13 ± 0.29 | 1.13 ± 0.32 | 1.11 ± 0.28 |
| 125 | 12.63 ± 2.01 | 3.02 ± 0.44 | 8.95 ± 4.12 | 12.93 ± 6.08 | 1.64 ± 0.35 | 4.2 ± 0.93 | 1.63 ± 0.21 |
| 250 | 6.96 ± 0.73 | 2.31 ± 0.3 | 8.84 ± 3.31 | 5.27 ± 2.3 | 1.54 ± 0.23 | 2.64 ± 0.41 | 1.47 ± 0.28 |
| 500 | 22.93 ± 9.07 | 3.43 ± 0.66 | 6.89 ± 2.28 | 5.37 ± 0.96 | 1.2 ± 0.22 | 8.48 ± 1.67 | 3.95 ± 0.82 |
| 1000 | 32.84 ± 21.02 | 34.69 ± 20.77 | 28.09 ± 6.7 | 3.18 ± 0.51 | 4.02 ± 1.8 | 100.42 ± 58.11 | 9.72 ± 2.18 |
| Lung gene expression: Sensitization Exposure | | | | | | | |
| 0 | 1.08 ± 0.22 | 1.04 ± 0.14 | 1.11 ± 0.25 | 1.08 ± 0.22 | 1.04 ± 0.14 | 1.23 ± 0.41 | 1.12 ± 0.25 |
| 125 | 1.95 ± 0.76 | 0.44 ± 0.11 | 0.54 ± 0.15 | 0.74 ± 0.2 | 0.8 ± 0.13 | 0.86 ± 0.15 | 0.83 ± 0.11 |
| 250 | 2.98 ± 0.82 | 1.07 ± 0.09 | 1.15 ± 0.22 | 0.95 ± 0.12 | 1.07 ± 0.11 | 0.79 ± 0.1 | 0.44 ± 0.07 |
| 500 | 1.21 ± 0.22 | 1.62 ± 0.36 | 1.3 ± 0.29 | 1.45 ± 0.26 | 1.44 ± 0.21 | 1.83 ± 0.4 | 1.98 ± 0.52 |
| 1000 | 1.56 ± 0.22 | 1.17 ± 0.19 | 1.22 ± 0.36 | 1.79 ± 0.37 | 2.07 ± 0.57 | 2.57 ± 0.54 | 3.44 ± 1.83 |
| Lung gene expression: Sensitization/Challenge Exposure | | | | | | | |
| 0 | 1.12 ± 0.25 | 1.25 ± 0.45 | 1.09 ± 0.23 | 1.07 ± 0.19 | 1.16 ± 0.35 | 1.06 ± 0.18 | 1.16 ± 0.35 |
| 125 | 0.9 ± 0.22 | 1.14 ± 0.12 | 1.02 ± 0.14 | 0.9 ± 0.16 | 0.98 ± 0.1 | 0.92 ± 0.12 | 0.91 ± 0.24 |
| 250 | 0.78 ± 0.1 | 0.89 ± 0.17 | 1.64 ± 0.75 | 0.69 ± 0.12 | 0.95 ± 0.17 | 1.07 ± 0.2 | 0.95 ± 0.37 |
| 500 | 2.97 ± 1.07 | 2.51 ± 0.77 | 1.81 ± 0.42 | 1.37 ± 0.4 | 1.52 ± 0.21 | 2.29 ± 0.89 | 0.4 ± 0.19 |
| 1000 | 1.88 ± 0.35 | 2.2 ± 0.71 | 2.85 ± 0.9 | 1.66 ± 0.25 | 1.28 ± 0.11 | 1.55 ± 0.18 | 1.22 ± 0.53 |

Cells highlighted in gray are significantly different from the concurrent control (0 ppb) group at *P* < 0.05

*representative data from one of the control groups

^a^Mean ± SEM (n=5)

Table S2: Absolute numbers of lymphocyte populations in the draining lymph nodes of mice following inhalation of OPA.

| **ppb** | **Total lymphocytes** | **B-lymphocytes** | **T-lymphocytes** | **CD4^+^ T-lymphocytes** | **CD8^+^ T-lymphocytes** |
| --- | --- | --- | --- | --- | --- |
| Sensitization Exposure | | | | | |
| 0 | 3.07 ± 0.55 | 1.02 ± 0.25 | 1.79 ± 0.26 | 0.8 ± 0.13 | 0.95 ± 0.12 |
| 125 | 11.8 ± 1.19 | 3.25 ± 0.49 | 7.64 ± 0.61 | 2.95 ± 0.26 | 4.58 ± 0.33 |
| 250 | 5.48 ± 0.61 | 2.09 ± 0.3 | 3.01 ± 0.34 | 1.34 ± 0.15 | 1.59 ± 0.19 |
| 500 | 15.51 ± 1.74 | 8.81 ± 1.31 | 5.75 ± 0.47 | 2.44 ± 0.21 | 3.05 ± 0.26 |
| 1000 | 26.89 ± 1.47 | 16.7 ± 0.78 | 7.42 ± 0.61 | 2.73 ± 0.22 | 4.51 ± 0.38 |
| Sensitization/Challenge Exposure | | | | | |
| 0 | 5.85 ± 0.92 | 2.23 ± 0.27 | 3.4 ± 0.58 | 1.45 ± 0.25 | 1.87 ± 0.31 |
| 125 | 10.95 ± 1.22 | 6.53 ± 0.79 | 3.88 ± 0.38 | 1.5 ± 0.14 | 2.27 ± 0.23 |
| 250 | 18.12 ± 1.95 | 8.61 ± 1.2 | 8.32 ± 0.88 | 3.37 ± 0.32 | 4.73 ± 0.54 |
| 500 | 10.26 ± 1.63 | 6.14 ± 1.04 | 3.86 ± 0.57 | 1.6 ± 0.25 | 2.15 ± 0.3 |
| 1000 | 20.71 ± 1.38 | 14.78 ± 1.03 | 4.8 ± 0.43 | 1.81 ± 0.22 | 2.84 ± 0.21 |

Cells highlighted in gray are significantly different from the concurrent control (0 ppb) group at *P* < 0.05

*represents data from one of the control groups

^a^Mean ± SEM (n=5)
